# A Modified Fluorimetric Method for Determination of Hydrogen Peroxide Using Homovanillic Acid Oxidation Principle

**DOI:** 10.1155/2014/342958

**Published:** 2014-05-19

**Authors:** Biswaranjan Paital

**Affiliations:** ^1^Biochemistry Unit, Department of Zoology and Biotechnology, Utkal University, Bhubaneswar 751004, India; ^2^Department of Molecular Reproduction, Development and Genetics, Indian Institute of Science, Bangalore 560012, India

## Abstract

Hydrogen peroxide (H_2_O_2_) level in biological samples is used as an important index in various studies. Quantification of H_2_O_2_ level in tissue fractions in presence of H_2_O_2_ metabolizing enzymes may always provide an incorrect result. A modification is proposed for the spectrofluorimetric determination of H_2_O_2_ in homovanillic acid (HVA) oxidation method. The modification was included to precipitate biological samples with cold trichloroacetic acid (TCA, 5% w/v) followed by its neutralization with K_2_HPO_4_ before the fluorimetric estimation of H_2_O_2_ is performed. TCA was used to precipitate the protein portions contained in the tissue fractions. After employing the above modification, it was observed that H_2_O_2_ content in tissue samples was ≥2 fold higher than the content observed in unmodified method. Minimum 2 h incubation of samples in reaction mixture was required for completion of the reaction. The stability of the HVA dimer as reaction product was found to be >12 h. The method was validated by using known concentrations of H_2_O_2_ and catalase enzyme that quenches H_2_O_2_ as substrate. This method can be used efficiently to determine more accurate tissue H_2_O_2_ level without using internal standard and multiple samples can be processed at a time with additional low cost reagents such as TCA and K_2_HPO_4_.

## 1. Introduction

Hydrogen peroxide (H_2_O_2_) is a major toxic byproduct of oxygen metabolism in both animals and plants. Its level therefore is used as a major biomarker in multiple investigations [1]. H_2_O_2_ reacts with superoxide radicals in Haber-Weiss or Fenton reaction and becomes the precursor for production of the highly toxic hydroxyl radical. H_2_O_2_ is also produced in other enzymatic reactions. It is toxic to the living cells in the range of 10–100 *μ*M [2, 3]. In context dependent manner, H_2_O_2_ level in tissues is used to monitor the effect(s) of various pharmacological and other substances for determining free radical generation or free radical scavenging activity [4]. H_2_O_2_ level is also correlated with several enzyme assays or levels such as superoxide dismutase (SOD), catalase (CAT), horse radish peroxidase (HRP), and lipoxygenase (LIP) which are involved in metabolism of H_2_O_2_ [5, 6]. Apart from these enzymes, xanthine oxidase, urate oxidase, and D-amino oxidase are also responsible for generating H_2_O_2_ in vivo [2]. Therefore, accurate measurement of H_2_O_2_ in tissues is important to determine its ecotoxic, physiological or other relevance(s) [7].

Although modern techniques such as flow cytometry, confocal microscopy and spin trapping methods are available to quantify reactive oxygen species in intact cell samples, biochemical quantification of H_2_O_2_ in tissue fractions is still used in many laboratories across the world [8]. However, the presence of enzymes such as SOD, CAT, LIP, HRP, and other peroxidases and oxidases in biological samples especially in tissue fractions may have a lot of interference during biochemical quantification of H_2_O_2_ [9]. Such enzymes can metabolize H_2_O_2_ during homogenization and centrifugation and during the time of incubation of samples in assay mixture for H_2_O_2_ estimation. Differential concentrations or activities of the above interfering enzymes in the tissue homogenate/lysate may lead to inaccurate H_2_O_2_ quantification. Most of the times, use of an internal standard is suggested to measure the exact tissue H_2_O_2_ content. However, internal standard cannot minimize the error when the interfering agents such as CAT and SOD are in highly variable quantities in samples [10]. Theoretically, it seems that the accurate value of H_2_O_2_ cannot be measured in tissue fractions when it contains its catabolizing enzyme, that is, CAT, having high Km value.

Using different substrates such as ferric-xylenol orange, homovanillic acid (HVA), scopoletin,* p*-hydroxyphenylacetic acid,* p*-hydroxyphenylpropionic acid, and tyramine, different biochemical methods are proposed to determine H_2_O_2_ content in tissues [4, 11, 12]. However, HVA-HRP system is widely used due to the stability of its fluorescent product (HVA dimer) being more than others such as scopoletin method [4]. Nevertheless, none of the biochemical methods describe any modification to remove H_2_O_2_ metabolizing enzymes from tissue fractions prior to H_2_O_2_ quantification. During our investigation on mud crabs, we encountered a similar problem to quantify H_2_O_2_ [13]. For the first time, a modified method is reported to estimate more accurate H_2_O_2_ content in biological samples. To achieve the objective, tissue homogenates were precipitated with trichloroacetic acid (TCA, 5% w/v, which precipitates proteins) and then were neutralized with K_2_HPO_4_ followed by H_2_O_2_ quantification. The method was validated using known concentrations of CAT and H_2_O_2_. Using this modification, multiple samples at a time can be processed to determine more accurate tissue H_2_O_2_ level with additional low cost reagents such as TCA and K_2_HPO_4_. Secondly, the final reaction product can be measured conveniently within 12 h without any significant loss of the fluorescent value.

## 2. Materials and Methods

### 2.1. Chemicals

Reagents from Sigma Chemical Company, USA, such as HRP, HVA (4-hydroxy-3-methoxyphenylacetic acid), tris base, and SOD were procured locally. TCA, K_2_HPO_4_ and KH_2_PO_4_ were purchased from SISCO Research Laboratory, Mumbai, India. H_2_O_2_ was obtained from SD Fine Chemicals, Mumbai, India. All other chemicals used were of the analytical grades.

### 2.2. Instrumentation

All fluorescence studies were performed at 25°C with a spectrofluorimeter (Hitachi, Model F 2500, Japan) using 1 cm^2^ quartz cuvette. Instrument specifications during measurements were 5 nm for both extinction and emission slit widths and 400 PMT voltages and 315 nm and 425 nm for extinction and emission wave lengths, respectively. A Varian CARRY 100 UV Visible spectrophotometer was used to quantify H_2_O_2_ at 340 nm wave length and for spectral analysis.

### 2.3. Principle and Procedure

**Figure 1:**
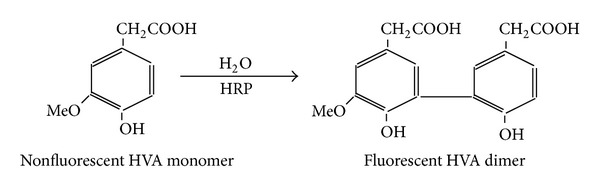
Mechanism of fluorescent dimer production of HVA after oxidation by H_2_O_2_ catalyzed by HRP.

The assay was based on H_2_O_2_ and HRP mediated oxidation of HVA (nonfluorescent) for the production of biphenyl HVA dimer which is fluorescent in nature. The HVA dimer is produced by a covalent bond formed by demolishing the methoxy group of the carbon atom of one HVA molecule at 3′ position with the carbon molecule at 5′ position of the other HVA monomer (Figure 1). The specificity of the assay is described elsewhere [14]. However, the spectral change that takes place after and before the reaction was also checked to confirm the HRP mediated oxidation of HVA by H_2_O_2_ and HRP system.

Reaction mixture for the calibration curve of H_2_O_2_ contained 1.4 mL of 25 mM phosphate buffer pH 7.6 (PB) with 0.2 mL HRP (0.1 U mL^−1^) and 0.2 mL HVA (125 *μ*M). Reaction was started with addition of 0.2 mL of tissue sample or H_2_O_2_ of required concentration into the reaction mixture. Similarly, appropriate blanks [(−H_2_O_2_ + HVA + HRP), (+H_2_O_2_+ HVA − HRP) and (+H_2_O_2_−HVA + HRP )] were run to check any background fluorescence of the reaction mixtures. Time kinetics was performed to determine the optimum time period for completion of the reaction at which all the H_2_O_2_ molecules were supposed to consume by the reaction system. Since the aim of the experiment was to precipitate the tissue samples with TCA, therefore, the required concentration of H_2_O_2_ was precipitated in TCA (5%, w/v) and then was neutralized with 6 M K_2_HPO_4_ (3.2 mL 6 M K_2_HPO_4_ with 15 mL H_2_O_2_ in 5% TCA) to keep the pH of the mixture within 7.50 ± 0.04. Then the fluorescence of the above TCA treated H_2_O_2_ was determined. H_2_O_2_ was also incubated with CAT enzyme (5–20 U) for different time periods (5–20 min) to confirm the role of H_2_O_2_ in the reaction system. After incubation with or without CAT, H_2_O_2_ samples were precipitated with TCA (5%) and then neutralized with required volume of K_2_HPO_4_ to keep the pH ~7.5. The reaction product of HVA dimer in both TCA treated and nontreated H_2_O_2_ samples was kept for different time periods (2–60 h) to check the stability of the HVA dimer product. Finally, the method was employed in light dependent superoxide generating system. The above was done to check H_2_O_2_ content by incubating the mixture with SOD enzyme which coverts superoxide anions to H_2_O_2_. Both tris and phosphate buffers were used to study the effects of buffers on the assay system. And there was no significant change in fluorescence pattern observed with either of the buffers. Therefore, the assay was carried out in phosphate buffer.

### 2.4. H_2_O_2_ Determination in Crab Tissues

It was observed that tissues of the crab* Scylla serrata* contain variable amount of H_2_O_2_ and CAT [13]. Also tissues of* S. serrata* contain other antioxidant enzymes such as SOD and glutathione peroxidase which metabolize H_2_O_2_ [15]. Along with the above reasons, as* S. serrata* is a good model to study several ecophysiological phenomenons [16], tissues of* S. serrata* were chosen for the present study to validate the method of modified H_2_O_2_ determination. Male* S. serrata* (65 ± 5 g) were obtained from the local market of Bhubaneswar, Odisha, India. Gill, hepatopancreas, and muscle tissues were dissected immediately after sacrificing the animals. 20% tissue homogenates of muscle, gill, and hepatopancreas were prepared in 25 mM PB containing 2 mM EDTA and Triton X-100 (0.1%, v/v), pH 7.6, using a motor driven glass-Teflon homogenizer at 4°C. Half of the crude homogenates were immediately precipitated with TCA (5%, w/v) and the rest of the halves were treated as the respective control. The volume used for addition of TCA into the sample was replaced with the volume with 25 mM PB for control samples. Both the homogenates were then centrifuged at 10,000 ×g for 10 min at 4°C in dark. The obtained supernatant was neutralized with the required quantity of 6 M K_2_HPO_4_ (214 *μ*L 6 M K_2_HPO_4 _mL^−1^ of sample in 5% TCA) and its H_2_O_2_ content was determined. The nonprecipitated samples were also subjected to the same procedure except PB was used in all steps instead of TCA and K_2_HPO_4_. Effort was made to avoid light exposure at each step since H_2_O_2_ is light sensitive. H_2_O_2_ was measured in both the samples and the fluorescence values were then compared. In case of crab hepatopancreas, the required dilution was done before using the samples for H_2_O_2_ content determination. Appropriate blanks [(−sample + HVA + HRP), (+sample + HVA − HRP) and (+sample −HVA + HRP)] were run to check any background fluorescence of the reaction mixtures. In the above step, both TCA precipitated and nonprecipitated samples were used.

### 2.5. Statistical Analyses

Data are presented as mean (± S.D.) of three samples each having technical triplicates. Student's *t*-test at *P* < 0.05 level was used to compare the values between TCA precipitated and control samples. One-way ANOVA followed by Duncan New Multiple range test was performed to validate the statistical difference between different time point groups of either TCA treated or control samples at *P* < 0.05.

## 3. Results

### 3.1. The Assay System

**Figure 2:**
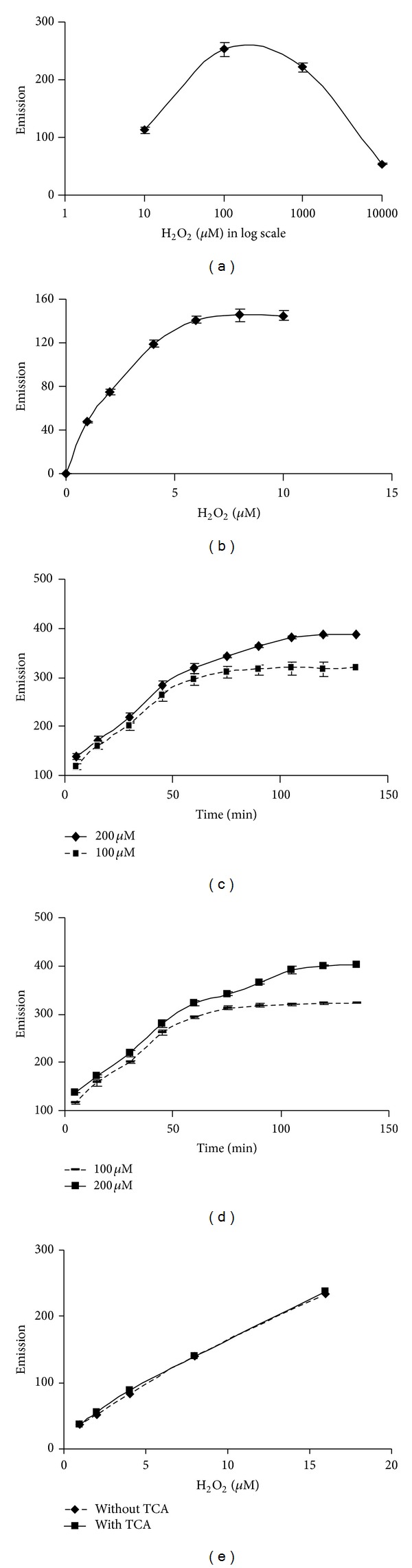
Calibration curves of H_2_O_2_ in the assay system. H_2_O_2_ concentration was used in the range of 1 to 1000 *μ*M (a) and 1–15 *μ*M (b) with 1 h incubation of the reaction mixture. Time kinetics (0–2 h) for completion of reaction for two concentrations of H_2_O_2_ (100–200 *μ*M) without (c) and with (d) TCA treatment of H_2_O_2_ followed by neutralization prior to use. Dose curve for H_2_O_2_ (1–16 *μ*M) in both TCA treated and nontreated condition with 2 h incubation of the reaction mixture (e). In all cases 125 *μ*M HVA and 0.1** **U/mL HRP were used in 2 mL reaction mixture at 25°C.

The dose curve was bell shaped when H_2_O_2_ was used in log phase concentrations from 1 *μ*M to 10, 000 *μ*M (Figure 2(a)). This broad range of H_2_O_2_ concentration was used to have a broad idea about the linearity of the dose curve of H_2_O_2_ with the given HVA and HRP concentration. Results of the present study reflect that calibration curve of H_2_O_2_ in 2 mL assay mixture (1–6 *μ*M of H_2_O_2_, 125 mM HVA, and 0.1 U mL^−1^ HRP) was linear when incubated for 1 h. However, the time required for the saturation of the reaction with the above assay composition appeared to be more than 1 h (Figures 2(b), 2(c), and 2(d)). Therefore, the actual time needed to have a maximum difference in emission value (in order to confirm the completion of the reaction) between two different concentrations of H_2_O_2_ (100 *μ*M–200 *μ*M) was determined. It was observed that around 2 h was needed to quench maximum number of the H_2_O_2_ molecules present in the reaction mixture with 125 *μ*M HVA and 0.1 U mL^−1^ HRP (Figures 2(c) and 2(d)). After 2 h incubation, no significant increase in the emission for both the concentrations of H_2_O_2_ was observed (Figure 2(c)) due to saturation of the assay system.

### 3.2. Effects of TCA Precipitation on the Assay System

**Figure 3:**
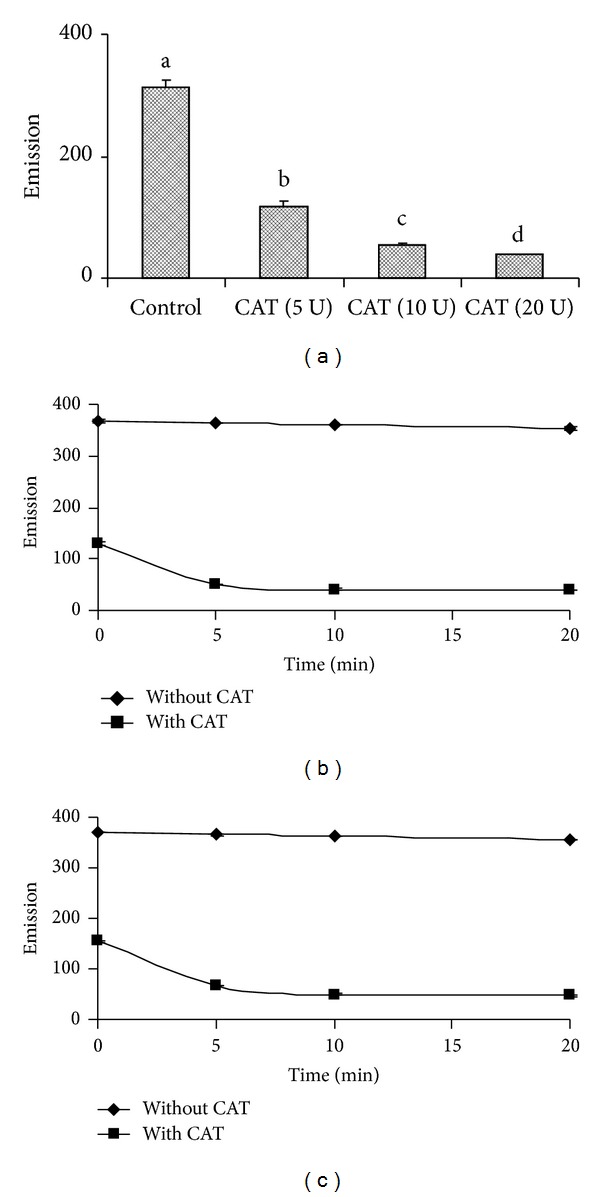
Estimation of H_2_O_2_ in the presence of catalase. (a) 200 *μ*M H_2_O_2_ was incubated with different concentrations of CAT for 10 min. Validation of the method with CAT and 200 *μ*M H_2_O_2 _treated with (c) or without (b) TCA. TCA treated H_2_O_2_ sample was neutralized with K_2_HPO_4_ before it was incubated with 20 U CAT followed by quantification. Results were the same regardless of using TCA. Different superscripts in Figure 3(a) indicate statistical significance at *P* ≤ 0.04.

**Figure 4:**
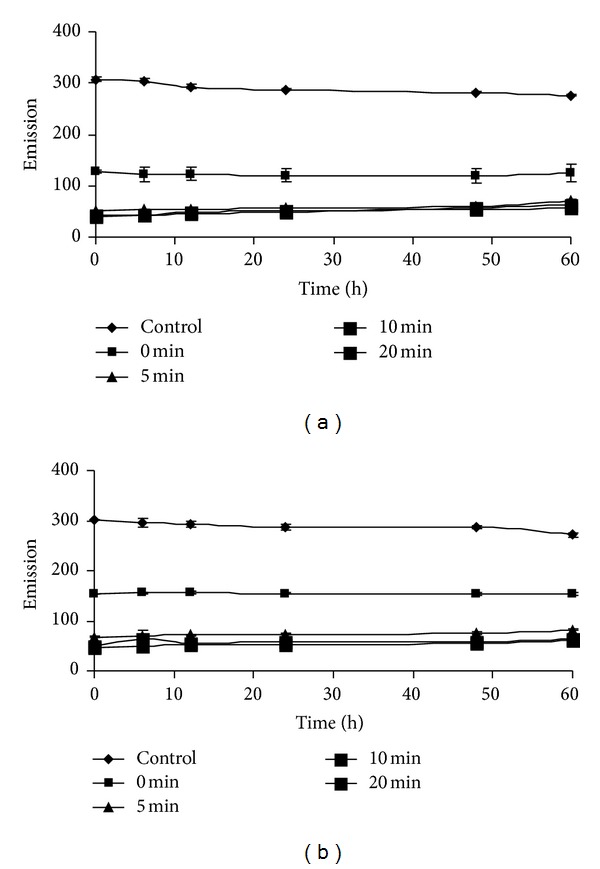
Stability of HVA dimer as reaction product in TCA treated (a) and TCA nontreated (b) H_2_O_2_ sample as a function of time. CAT (20 U) was incubated with the reaction mixture for different time periods (0–20 min) followed by TCA (5%, w/v) precipitation, neutralization with K_2_HPO_4_, and H_2_O_2_ quantification in HVA-HRP method.

**Figure 5:**
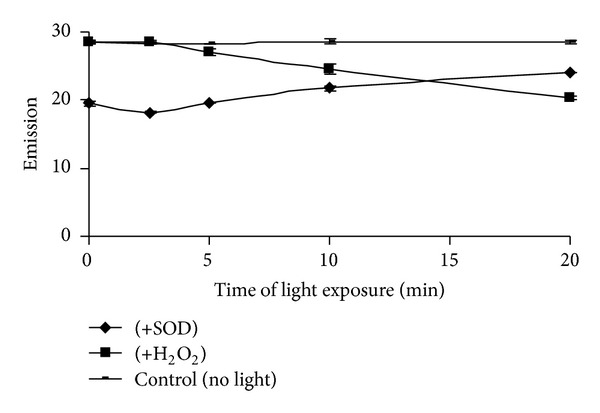
Failure of the HRP-HVA method for H_2_O_2_ quantification in light dependent H_2_O_2_ production system. Superoxide radicals were generated in light dependent riboflavin reduction system and were converted to H_2_O_2_ by the enzyme SOD (20 U mL^−1^). H_2_O_2_ (2 *μ*M) was used as positive control in presence or absence of light.

Dose curve of TCA precipitated H_2_O_2_ (1–16 *μ*M) followed by neutralization with K_2_HPO_4_ was not only similar but also was almost the same with that of their corresponding control samples (Figure 2(e)). The saturation time for completion of the reaction in the above conditions for 100 *μ*M and 200 *μ*M H_2_O_2_ in both TCA precipitated and nonprecipitated condition was found to be 2 h (Figures 2(c) and 2(d)). CAT uses H_2_O_2_ as its substrate. Almost linear decreased emission value was observed when the assay mixtures were preincubated with different concentrations (5–20 U) of catalase enzyme for different (0–20 min) time periods (Figures 3(a), 3(b), and 3(c)). As H_2_O_2_ with CAT after precipitation with TCA did not show further increase in fluorescence value, therefore, it was confirmed that CAT got precipitated with 5% TCA (Figure 4). The HVA dimer product in both TCA treated and nontreated H_2_O_2_ was found to be stable up to at least 12 h. However, a 10% loss in fluorescence of the HVA dimer, produced after the end of the reaction was observed after 60 h than 2 h (Figure 4). Das et al. [17] described a method in which superoxide radical is produced by photoreduction of riboflavin and subsequently measured by spectrophotometric method. SOD converts superoxide radical to H_2_O_2_. Using the above principles, they implemented the measurement system finally for determination of SOD content in samples. Logically it was expected in the above system that if SOD will be added instead of tissue samples then the produced superoxide radicals can be converted into H_2_O_2_ and then can be measured using the current principle of HVA oxidation method. The results depicted that H_2_O_2_ cannot be measured by this method where light is a precursor for its production such as in superoxide radical generating system by riboflavin reduction method [17] incubated with SOD enzyme (Figure 5).

### 3.3. Validation of the Method in Biological Samples

**Figure 6:**
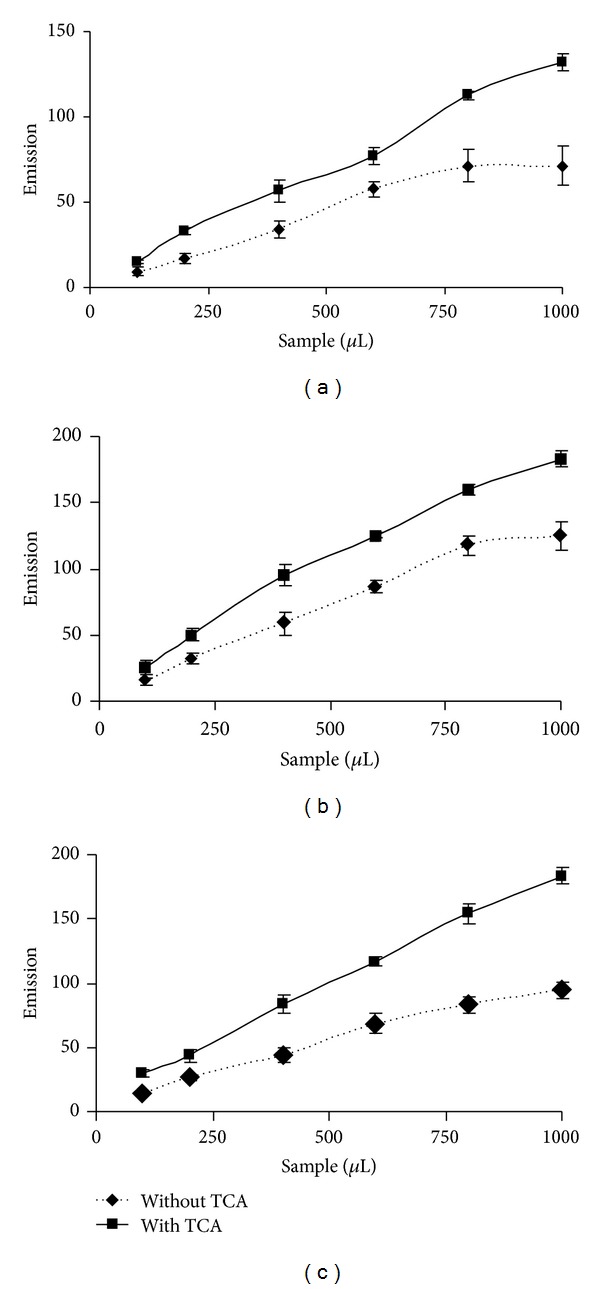
Determination of H_2_O_2_ in tissue fractions of the mud crab* Scylla serrata. *In muscle (a), gills (b), and hepatopancreas (c), the fluorescence of the TCA treated samples was almost ≥ 2 folds higher than its corresponding nontreated sample. Tissue fractions were precipitated immediately after homogenization followed by neutralization with K_2_HPO_4_ and H_2_O_2_ quantification.

The above modification of HVA oxidation method to determine H_2_O_2_ content in samples was tested in a crab model. In TCA treated muscle, gills, and hepatopancreas tissue fractions of the crab* S. serrata*, a minimum of 2.0, 2.2, and 2.5 folds (*P* < 0.05) higher amount of H_2_O_2_ was observed than TCA non-treated samples (Figure 6).

## 4. Discussion

H_2_O_2_ in tissues is used as a major biomarker in multiple biochemical investigations [1]. H_2_O_2_ mediated production of highly toxic hydroxyl radical has also many biochemical implications [3]. H_2_O_2_ is metabolized in many enzymatic reactions in tissues. Therefore, to quantify the accurate level of H_2_O_2_ in tissues (containing its metabolizing enzymes) using biochemical methods can be a challenge. A modified method using HVA oxidation principle is proposed to measure the exact H_2_O_2_ level in tissues. Homovanillic acid is a nonfluorophore compound which is converted to a highly fluorescent compound after oxidation in HRP-H_2_O_2_ system (Figure 1). This principle is used to estimate H_2_O_2_ in biological samples [14]. In all assays where H_2_O_2_ is quantified by employing HVA-HRP system, the reaction mixture is incubated for different time intervals ranging from 5 min to 1 h [4, 6, 18]. Then the final fluorescent products are measured without terminating the reaction. Efforts had also been made to terminate the reaction by increasing the pH of the reaction mixture to alkaline range (pH > 10) before estimating the fluorescent intensity of the samples [18]. In both of the above cases, it seems that, without determining the time kinetics for saturation of the reaction, accurate amount of H_2_O_2_ in the reaction mixture may not be determined. This is because a certain amount of H_2_O_2_ may be retained in the reaction mixture due to insufficient time to end the reaction (at high pH) before it is being completed. Most of the biological samples especially tissue fractions contain several enzymes such as CAT and several peroxidase and oxidase enzymes which can metabolize H_2_O_2_. During tissue fractionation and incubation steps for H_2_O_2_ determination in such samples, the possibility of getting inconsistent result cannot be ruled out. Therefore, the assay system can measure more accurate H_2_O_2_ content in the sample if only the H_2_O_2_ metabolizing enzymes are removed from the reaction mixture and the reaction mixture gets sufficient time period for completion. In this communication, a small but important modification in HVA oxidation method to estimate H_2_O_2_ in biological samples is reported. This was achieved by precipitating tissue fractions with ice cold TCA (5%) and neutralization with K_2_HPO_4_ followed by H_2_O_2_ quantification. The time kinetics of the reaction to determine the optimum time required to complete the reaction between all H_2_O_2_ and HVA molecules in the system was also investigated.

Decreased florescence values that were observed for H_2_O_2_ samples with CAT treatment suggest the specificity of the reaction by H_2_O_2_ only. Time kinetics study suggests that minimum 2 h incubation time of assay mixture was required (1 to 25 *μ*M range of H_2_O_2_ or sample with 125 *μ*M HVA and 0.1 U mL^−1^ HRP) to complete the reaction. However, one can reduce the time of incubation by increasing the HRP and HVA concentration [4, 19]. HVA dimer as the reaction product in both TCA treated and nontreated pure H_2_O_2_ and tissue samples was found to be stable for more than 60 h with only loss of 10% of fluorescence intensity. However, up to 12 h, the loss was less than 2% and statistically was not significant than the value observed after 2 h incubation. Thus, reaction product can be kept conveniently in the dark for at least half a day for quantification of H_2_O_2_.

An attempt was made to measure superoxide radical (having very low half-life) by employing this method. Light mediated riboflavin reduction method was employed for superoxide generation. SOD enzyme converts superoxide radicals to H_2_O_2_. The above assay mixture that generates superoxide radical under light was incubated with SOD enzyme. It was expected that H_2_O_2_ could be measured and the value could be correlated with the generated superoxide radical level. However, no significant fluorescent value was obtained in the process for H_2_O_2_ in the above system. It may be due to the breakdown of the produced H_2_O_2_ under high fluorescent light.

Finally, H_2_O_2_ content in the tissues of an invertebrate containing most of the H_2_O_2_ metabolizing enzymes (the mud crab* S. serrata*) was measured. It was observed that around ≥2 folds H_2_O_2_ was lost in muscle, hepatopancreas, and gills of the crab during tissue processing and incubation time for H_2_O_2_ determination. Recently, it is described that activities of CAT and glutathione peroxidase, the two major H_2_O_2_ catalyzing enzymes in this crab, are tissue-specific [20]. This may be the cause for tissue-specific loss of H_2_O_2_ in the crabs during H_2_O_2_ determination without TCA precipitation. Therefore, the loss of H_2_O_2_ during measurement may vary in different organs of an organism and in the same organ of different organisms due to the difference in H_2_O_2_ contents and H_2_O_2_ metabolizing enzymes.

One of the limitations of this method was that tissues were not directly homogenized in acids such as metaphosphoric acid which is used to remove enzymes from tissue fractions. TCA is an aggressive acid and, therefore, homogenization of tissues directly in TCA is not recommended. Neutralization of TCA (5%, w/v) treated samples was needed to keep the pH between 7 and 8 because HRP has optimum activity within this pH range. Therefore, the exact stoichiometry of the neutralization reaction between TCA (5%, w/v) and K_2_HPO_4_ (6 M) was not evaluated. So, the neutralization of TCA treated samples to study the final pH must be done prior to any assay. The method can also be applied to study other metabolites in tissue fractions where specific enzymes interfere in assaying a particular metabolite. However, before adapting TCA precipitation method to quantify a particular metabolite, its assay system must be checked in similar way with TCA and the pure metabolite. This modification method can be used in multiple studies where tissue H_2_O_2_ level is used as an important index [1]. The optimization of the HVA concentration and HRP activity may be carried out for further optimization of the method. Sometimes, much higher amount (>1 mL) of both TCA treated and untreated sample showed lower fluorescence values in comparison to the respective lower sample volumes. The reason may be attributed to the artifacts that quench or hinder the HVA dimer production.

It is claimed that phosphate ion or buffer, if used, may interfere in the fluorescence assay system [21]. However, the above authors used phosphate buffer to check the effects of pH (7–9.5) on the detection system using immobilized peroxidase with a fibre-optic detector. They observed that the naturalized pH was the optimum to get the fluorescence signal by the optic sensor. This could be due to the activity optima of immobilized HRP in neutralized pH. Apparently, no reports are available exclusively to check the effects of phosphate ion on the HRP and HVA mediated H_2_O_2_ assay system. However, it seems that there is a very less chance for the interference of phosphate ion on the assay system as no difference was observed in TCA treated and untreated positive controls, that is, pure H_2_O_2_ samples, because, in TCA treated pure H_2_O_2_ samples, high concentrations of phosphate ions would be there since 6 M K_2_HPO_4_ was used to neutralize it. Although higher fluorescence values were observed in the present study with tris buffer than phosphate, it was statistically not significant (data not given). Nevertheless, a negative control by using excess catalase in the tissue sample prior to TCA precipitation would enhance the accuracy of the method. However, no fluorescence was observed in the samples when intentionally the stored (at 4°C for 1-2) tissue fractions were used for H_2_O_2_ content measurement (data not given). The reason(s) could be contributed to the H_2_O_2_ scavenging activity of the tissue fractions due to multiple reasons including acceleration of Fenton chemistry and/or utilization of the H_2_O_2_ by the presence of internal catalase and other H_2_O_2_ catabolizing enzymes.

Shirasaka et al. [22] noticed that HRP also can break down lipid hydroperoxides and can suppress peroxidation of polyunsaturated fatty acids in the presence of phenolic antioxidants (in 100–200 *μ*M range) such as quercetin, capsaicin, and *α*-tocopherol but not in presence of the reduced glutathione. Therefore, the presence of the above phenolic antioxidants in *μ*M range to interfere in the HRP mediated HVA dimer production by H_2_O_2_ in crab samples is very low. Also for the above additional function of HRP, it needs a proper stoichiometry between HRP and the phenolic antioxidants (in *μ*M range) in the tissue fractions. Phenolic antioxidants are more specifically observed in plant tissues. On the other hand, the specificity of the HRP mediated HVA dimer production can also be evaluated from the basal level of the fluorescence values that were obtained for the blank (assay mixture having no tissue fractions), from samples having tissue fractions and HVA only, and from positive controls. The specificity of the HRP mediated HVA dimer production by H_2_O_2_ is also reported by several authors [6, 14] and, therefore, claiming the interference of the above additional function of HRP in the presently proposed assay system can be forfeited, because, due to the above additional function of HRP, its availability for the assay system would not be nullified; rather it would be a little less.

TCA precipitates proteins in biological samples. It is concluded that TCA precipitation can be employed to prevent internal loss of H_2_O_2_ during processing of tissue samples in order to measure more accurate tissue H_2_O_2_ content in samples. Further, several samples can be processed simultaneously for H_2_O_2_ determination and the end product conveniently can be measured within 12 h.
